# Calcium-deficient diet attenuates carbon tetrachloride-induced hepatotoxicity in mice through suppression of lipid peroxidation and inflammatory response

**DOI:** 10.1016/j.heliyon.2016.e00126

**Published:** 2016-06-24

**Authors:** Hiroki Yoshioka, Tsunemasa Nonogaki, Nobuyuki Fukuishi, Satomi Onosaka

**Affiliations:** a College of Pharmacy, Kinjo Gakuin University, 2-1723 Omori, Moriyamaku, Nagoya, Aichi 463-8521, Japan; b Faculty of Nutrition, Kobe Gakuin University, 518 Arise, Ikawadani-cho, Nishi-ku, Kobe, Hyogo 651-2180, Japan

**Keywords:** Food science, Pharmaceutical science, Cell biology, Physiology

## Abstract

The aim of this study is to investigate whether a Ca-deficient diet has an attenuating effect on carbon tetrachloride (CCl_4_)-induced hepatotoxicity. Four-week-old male ddY mice were fed a Ca-deficient diet for 4 weeks as a part of the experimental protocol. While hypocalcemia was observed, there was no significant change in body weight. The CCl_4_-exposed hypocalcemic mice exhibited a significant decrease in alanine aminotransferase and aspartate aminotransferase activities at both 6 h and 24 h even though markers of renal function remained unchanged. Moreover, lipid peroxidation was impaired and total antioxidant power was partially recovered in the liver. Studies conducted in parallel with the biochemical analysis revealed that hepatic histopathological damage was attenuated 24 h post CCl_4_ injection in hypocalcemic mice fed the Ca-deficient diet. Finally, this diet impaired CCl_4_-induced inflammatory responses. Although upregulation of Ca concentration is a known indicator of terminal progression to cell death in the liver, these results suggest that Ca is also involved in other phases of CCl_4_-induced hepatotoxicity, via regulation of oxidative stress and inflammatory responses.

## 1 Introduction

Carbon tetrachloride (CCl_4_) is widely used in experimental animal models that are meant to mimic human hepatotoxicity. The mechanism of CCl_4_ hepatotoxicity has been thoroughly studied since the 1970s, by using *in vivo* models of acute and chronic CCl_4_ poisoning, perfused livers, and isolated or cultured hepatocytes [1, 2]. CCl_4_-induced toxicity is a multifactorial process involving the generation of free radicals [2, 3, 4, 5]. The first step is the metabolic activation of CCl_4_ by CYP2E1. Subsequently, CCl_4_ is converted to free radicals (trichloromethyl and trichloromethyl peroxy radicals). The second step is radical binding; the free radicals react with antioxidant enzymes and sulfhydryl groups such as those in glutathione (GSH) and the protein thiol. The third step involves the overexpression of these free radicals leading to several deleterious effects such as enhanced membrane lipid peroxidation, covalent binding to macromolecules, ATP depletion, generation of inflammatory cytokines, and loss of Ca homeostasis [6, 7, 8]. Since sulfhydryl groups are essential elements of the molecular arrangement responsible for the Ca transport access cellular membranes, loss of these proteins inhibits microsomal and mitochondrial regulation of cellular Ca levels.

Cadmium (Cd) has been classified by the International Agency for Research on Cancer as a group I carcinogen and is a ubiquitous contaminant of the environment and dietary product. Exposure to Cd is known to cause hepatic injury in acute toxicity and renal injury in chronic toxicity [9]. Cd-related toxicity is also a multifactorial process [10, 11, 12, 13]. The first step of which is GSH depletion. GSH depletion raises the level of lipid peroxidation in the cell membrane and mitochondrial dysfunction occurs as the next step. In addition, after these actions, disruption of calcium homeostasis and calcium uptake is observed. These mechanisms indicated that calcium uptake is the terminal phase of cell death. However, *Acosta and Sorensen* reported that Cd-induced cytotoxicity is impaired in a Ca-free medium *in vitro* [14]. This phenomenon was also observed by our investigations (data not shown, manuscript in progress). These data suggests that Ca is directly involved in Cd-induced toxicity, not only in the terminal phase, but also in other phases. These can be considered closely similar to the phases of CCl_4_-induced toxicity. Therefore, we hypothesized that Ca could exacerbate CCl_4_-induced toxicity as well.

To address this, the current study was carried out to investigate whether hypocalcemia in mice decreases CCl_4_-induced toxicity or not. To examine this, we fed mice a Ca-deficient diet and determined plasma biochemical markers, hepatic lipid peroxidation, and the hepatic inflammatory response.

## 2 Material and methods

### 2.1 Animal treatment

Male ddY mice were purchased from Japan SLC (Shizuoka, Japan) at 3 weeks of age. The mice were maintained under standard conditions of controlled temperature (24 ± 1 °C), humidity (55 ± 5 %), and light (12:12 h light/dark cycles) with free access to water and food. After acclimatization to a normal diet (CE-2; Clea Japan, Inc., Tokyo, Japan; [protein (soybean waste, whitefish meal, yeast): 24.9 %; carbohydrate (wheat flour, corn, Milo): 51.0 %; fat (cereal germ, soybean oil): 4.6 %; Ca: 1.06 g; and the other: 3.59 kcal/g]) for 1 week, the mice were divided into 2 groups of 8 or 9 each. One group was fed the CE-2 diet, and the other group was fed a Ca-deficient diet based on AIN–93 G (Oriental Yeast Co., Tokyo, Japan) [protein (casein, L-cysteine): 20.0 %; carbohydrate (corn, maltodextrin, sucrose): 64.0 %; fat (soybean oil, t-butylhydroquinone): 7.0 %; Ca: 0 g; and the other: 3.90 kcal/g] for 4 weeks. Food intake was monitored and body weight was measured once per week throughout the study. We collected blood samples from each mouse every 2 weeks to confirm the effects of the Ca-deficient diet on plasma Ca concentrations. After a final plasma Ca determination at 8 weeks of age, each mouse was injected intraperitoneally (i.p.) with 2 g/kg (at 5 mL/kg) CCl_4_ (Wako Chemical, Osaka, Japan). After 6 h and 24 h, blood samples were collected from each group of mice. Whole blood was centrifuged (3000 g, 10 min) and the supernatant was tested for hepatic and renal injury markers. The liver from each animal was harvested 24 h after CCl_4_ injection, and separate samples were stored at −80 °C or fixed in 15 % neutral buffered formalin (pH 7.2). All experiments were approved by the Institutional Animal Care and Experiment Committee of Kinjo Gakuin University (NO.110).

### 2.2 Plasma biochemical analysis

Plasma Ca levels were measured using the calcium-E test (Wako Chemical) according to the manufacturer’s instructions. Plasma samples (2.5 μL) were mixed with the substrate buffer (100 μL) and a coloring reagent (50 μL). The absorbance of the reaction mixture was measured at 610 nm.

Plasma alanine aminotransferase (ALT) and aspartate aminotransferase (AST) activities were measured using the Transaminase CII Test Wako (Wako Chemical) according to the manufacturer’s instructions and as previously described [15, 16]. Concentrations of plasma creatinine and blood urea nitrogen (BUN) were measured using the Creatinine Liquid Reagents Assay (DIAZYME, Poway, CA, USA) and the BUN Wako Test (Wako Chemical), according to the manufacturer’s instructions and as previously described [17]. For relative quantification, calibration curves were prepared using a standard solution.

### 2.3 Measurement of thiobarbituric acid levels in the liver

The total liver thiobarbituric acid (TBA) levels and antioxidant power were examined by a colorimetric microplate assay (Oxford biochemical research, Oxford, MI, USA) according to the manufacturer's protocol and as previously described [16, 17].

### 2.4 Histopathological findings

For histological analysis, a portion of the left lobe of the liver from each animal was perfused with 15 % phosphate-buffered neutral formalin (pH 7.2: Wako Chemical), dehydrated, and embedded in paraffin. Embedded tissues were sectioned at 4 μm thickness and stained with hematoxylin and eosin (H&E) or periodic acid Schiff (PAS) using standard methodologies. Histopathological features of the slices were observed using a light microscope.

### 2.5 Isolation of total RNA and RT-PCR assay

Total RNA was extracted from 0.1 g liver sections using ISOGEN II (Nippon Gene, Tokyo, Japan). Quantitative RT-PCR was performed with the One Step SYBR PrimeScript PLUS RT-PCR kit (Perfect Real Time) (Takara Bio, Shiga, Japan) using an Applied Biosystems 7300 (Applied Biosystems, Foster City, CA). PCR was performed in a 20 μL of solution containing 0.4 μM primers, 0.4 μL ROX Dye, and sample RNA (30 ng) in 2X One Step SYBR RT-PCR Buffer 4, TaKaRa Ex Taq HS Mix and PrimeScript PLUS RTase Mix. PCR conditions were as follows: 42 °C for 5 min, 95 °C for 10 s, and 40 cycles of 95 °C for 5 s followed by 60 °C for 31 s. Primer pairs are shown in Table 1. Relative expression of each mRNA was determined using the standard curve method. The amount of each target mRNA quantified was normalized against that of GAPDH-encoding mRNA.

### 2.6 Measurement of hepatic tumor necrosis factor (TNF)-a level by enzyme-linked immunosorbent assay (ELISA)

Aliquots (0.1 g each, including mixed cell types) of hepatic tissue were homogenized in 900 μL ice-cold phosphate-buffered saline (PBS) containing a protease inhibitor (Nacalai Tesque, Kyoto, Japan) and centrifuged at 18000 g for 20 min at 4 °C. The resulting supernatant (diluted to yield consistent total protein concentrations) for each sample was used for further steps. Hepatic levels of TNF-α were determined using a commercially available ELISA kit (eBioscience, San Diego, CA, USA), according to the manufacturer’s instructions. TNF-α concentrations were determined from a standard curve, and were expressed as pg/mL.

### 2.7 Statistical analysis

All data from the control and treatment groups were obtained from the same numbers of replicated experiments. All experiments were performed independently at least twice. Comparisons between the two groups were made using Student’s *t* test or Welch’s *t* test and multiple comparisons were analyzed using One Way ANOVA with post-hoc Tukey-Kramer’s test. All statistical analyses were performed using SPSS 19.0J software (Chicago, IL, USA). Values of *P* < 0.05 were considered statistically significant.

## 3 Results

### 3.1 Effect of the Ca-deficient diet on biochemical markers and body weight in CCl_4_-induced toxicity

**Fig. 1:**
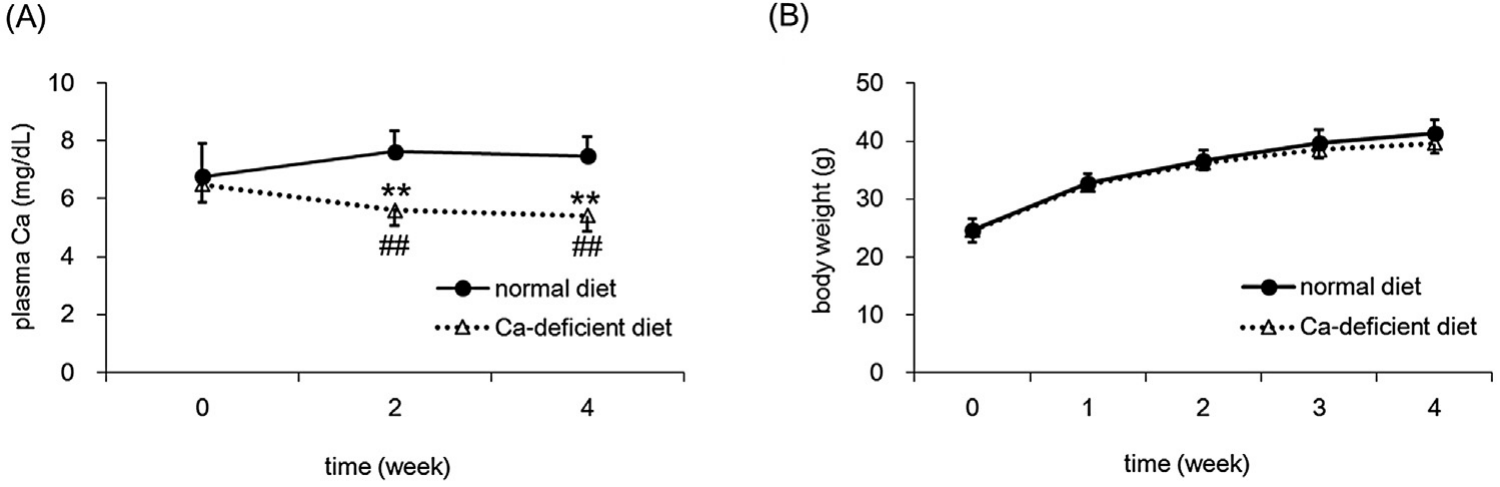
Effect of feeding a normal diet versus a Ca-deficient diet on body weight and plasma Ca concentration. Mice were fed a normal diet or a Ca-deficient diet from 4 weeks of age for a period of 4 weeks. Plasma Ca levels (A) were determined every 2 weeks and body weight change (B) was observed every week. Data indicate mean ± S.D. for 8–9 mice. ^**^P < 0.01 versus 0-week Ca-deficient diet group, ^##^*P < 0.01* versus normal diet group at the same week.

Plasma Ca concentrations decreased after 2 weeks of feeding and 28 % suppression was observed after 4 weeks (Fig. 1A). In addition to plasma Ca concentrations, the levels of ALT, AST (markers of hepatic injury), creatinine, and BUN (markers of renal injury) were compared between the normal diet and Ca-deficient diet groups (data not shown). In this study, no significant changes in body weight gain, or food intake were observed (Fig. 1B, Table 2).

### 3.2 Effect of the Ca-deficient diet on hepatic and renal injury markers in acute CCl_4_ toxicity

**Fig. 2:**
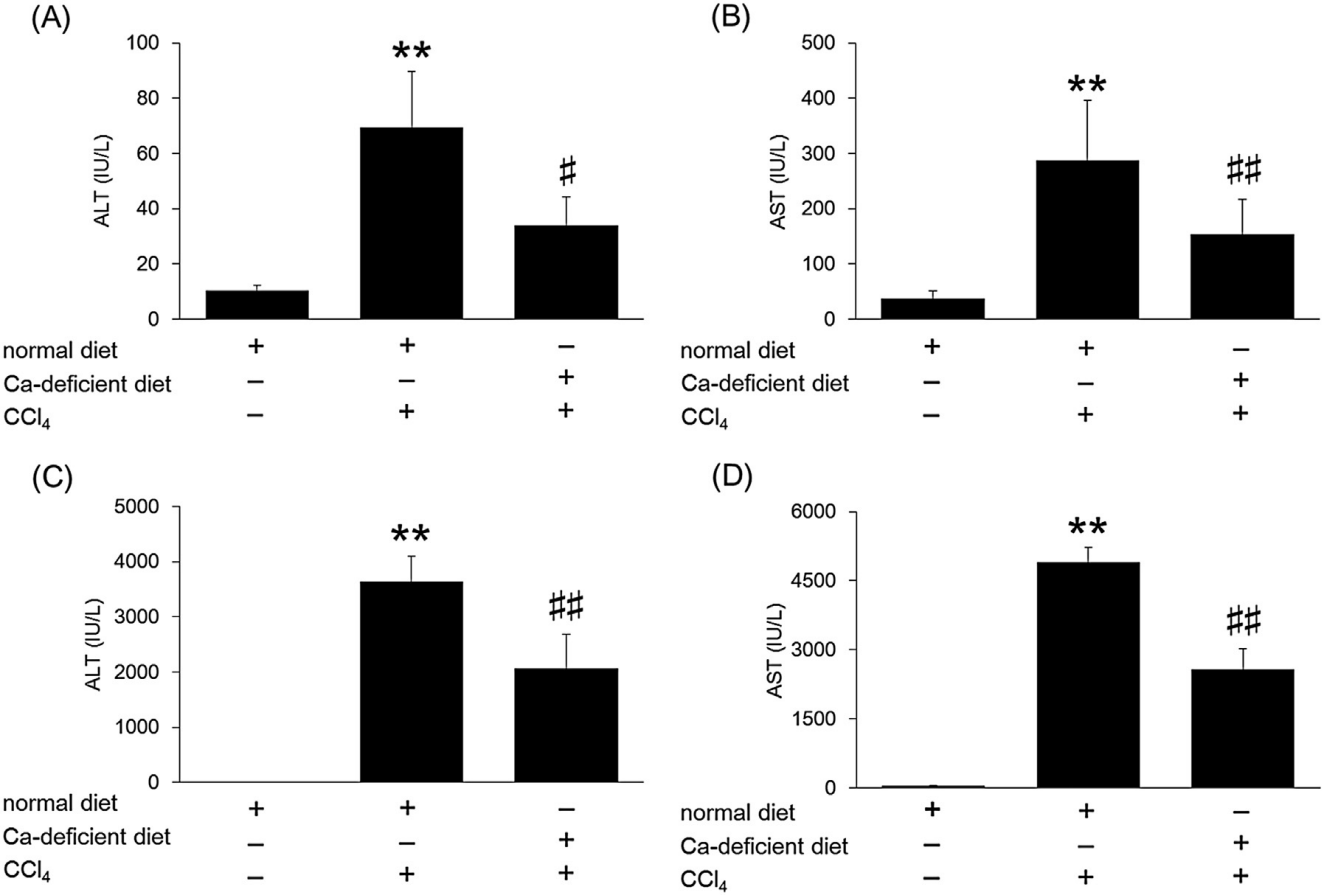
Effect of Ca-deficient diet on ALT and AST activities in CCl_4_-induced toxicity. Mice were fed a normal diet or a Ca-deficient diet from 4 weeks of age for a period of 4 weeks. At 8 weeks of age, mice were injected intraperitoneally with 2 g/kg CCl_4_. At 6 h (A and B) and 24 h (C and D) post CCl_4_ injection, plasma ALT (A and C) and AST (B and D) activities were determined. Data indicate mean ± S.D. for 6–9 mice. ^**^P < 0.01 versus control, ^#^*P < 0.05* and ^##^*P < 0.01* versus normal diet + CCl_4_ group.

To determine how CCl_4_-induced toxicity is impaired under hypocalcemic conditions, we examined hepatic injury markers ALT and AST, whose activities increase in CCl_4_-induced toxicity. As shown in Fig. 2, mice feeding on a Ca-deficient diet had significantly reduced ALT and AST activities at both 6 h and 24 h post injection.

In addition to ALT and AST, creatinine and BUN were also evaluated as markers of renal injury. As shown in Table 3, although CCl_4_ increases both creatinine and BUN, the levels of these parameters in our hypocalcemic mice were comparable.

### 3.3 Evaluation of Ca-deficient diet against CCl_4_ acute toxicity on TBA and total antioxidant levels in the liver

**Fig. 3:**
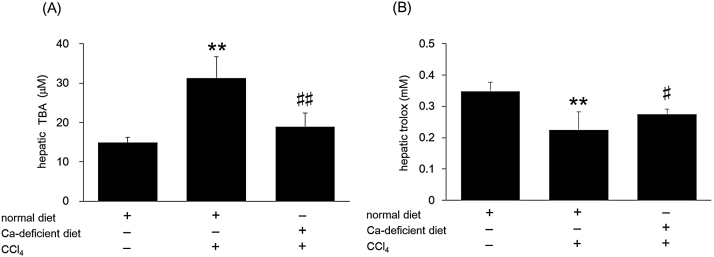
Effect of Ca-deficient diet on TBA levels and antioxidant power in CCl_4_-induced toxicity. Mice were fed a normal diet or a Ca-deficient diet from 4 weeks of age for a period of 4 weeks. At 8 weeks of age, the mice were injected intraperitoneally with 2 g/kg CCl_4_. TBA levels (A) and total antioxidant power (B) in the liver were determined 24 h after injection. Data indicate mean ± S.D. for 6–9 mice. ^**^P < 0.01 versus control, ^#^*P < 0.05* and ^##^*P < 0.01* versus normal diet + CCl_4_ group.

To further investigate hypocalcemia-induced attenuation of CCl_4_ liver toxicity, we measured TBA levels as a marker of lipid peroxidation. CCl_4_ treatment significantly increased hepatic TBA levels in mice on the normal diet while a partial reduction in the upregulated TBA levels was observed in mice on the Ca-deficient diet (Fig. 3A).

Studies suggest that total antioxidant power may be used as an indicator of oxidative stress levels. As shown in Fig. 3B, CCl_4_ treatment markedly decreased the total antioxidant power in the mouse liver. However, mice on a Ca-deficient diet recovered the lost antioxidant power.

### 3.4 Effect of Ca-deficient diet on CCl_4_-induced acute toxicity as assessed by hepatic structure

**Fig. 4:**
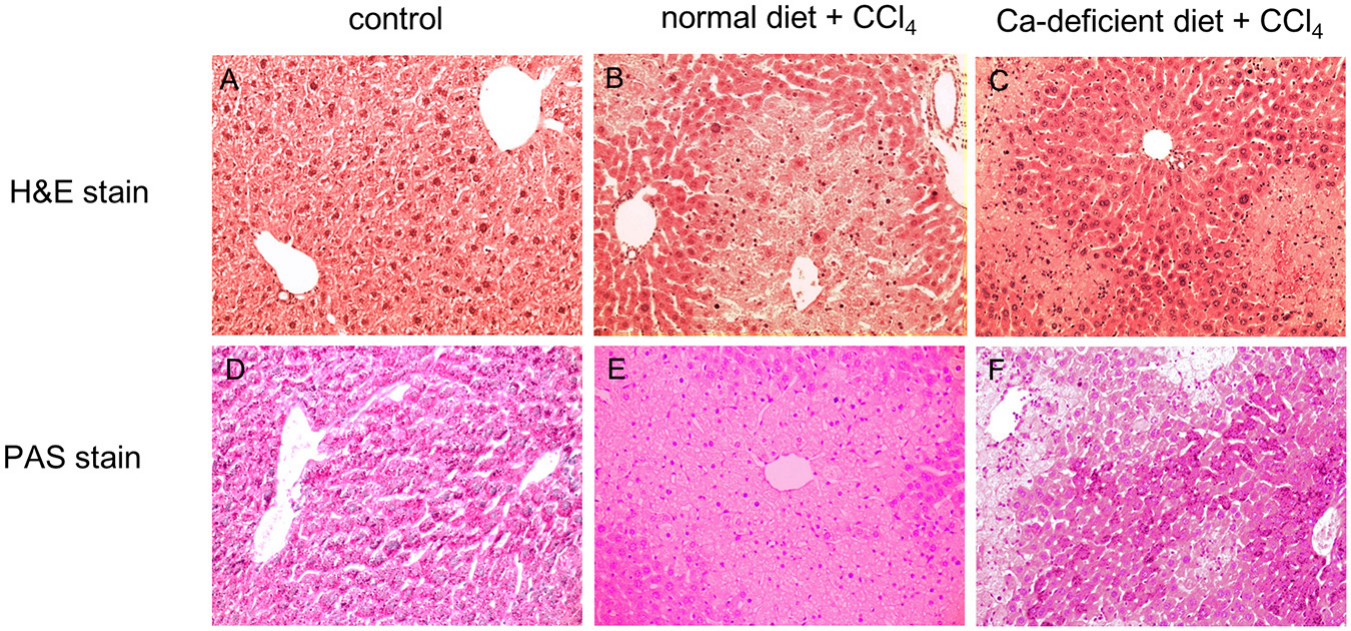
A Ca-deficient diet protects animals from acute CCl_4_-induced hepatotoxicity, as assessed by hematoxylin and eosin (H&E) and periodic acid Schiff (PAS) staining. Mice were fed a normal diet or a Ca-deficient diet from 4 weeks of age for a period of 4 weeks. At 8 weeks of age, the mice were injected intraperitoneally with 2 g/kg CCl_4_. Animals were euthanized at 24 h after the intraperitoneal injection and the livers were harvested at necropsy. Liver specimens were fixed and processed by standard methods, and sections were stained with H&E (A–C) or PAS (D–F). These micrographs provide 10x magnified images of representative H&E or PAS stained sections from livers obtained from control (A and D), normal diet + CCl_4_ (B and E), and Ca-deficient diet + CCl_4_ (C and F) animals. The image in (B) reveals severe necrosis around the central vein in CCl_4_ exposed animals fed a normal diet, in contrast to the mostly normal hepatic structure seen in (A) and (C). The image in (E) reveals almost complete depletion of hepatic glycogen following CCl_4_ intoxication while on a normal diet; in contrast, Ca-deficient diet group shows prevention of some of this glycogen depletion (F).

In parallel with the measurement of functional markers (Fig. 2 and Fig. 3, Table 3), we conducted histopathological studies. Liver sections obtained from the control group and stained with H&E showed normal cell morphology, well-preserved cytoplasm, and a clear, plump nucleus (Fig. 4A). The CCl_4_-injected mice on a normal diet showed signs of extensive necrosis (especially in the acinus, zone 3) (Fig. 4B), while those on a Ca-deficient diet counteracted some, but not all, of this liver necrosis (Fig. 4C). In the normal livers, glycogen granules accumulated diffusely in the hepatocytes, as shown by PAS staining (Fig. 4D). However, intrahepatic glycogen was almost completely depleted in the liver sections of CCl_4_-exposed mice in the normal diet group (Fig. 4E), while the livers of the CCl_4_-exposed animals in the Ca-deficient diet group recovered some hepatic glycogen content (Fig. 4F).

### 3.5 Estimation of hypocalcemic effect on CCl_4_-induced inflammatory response and CYP induction

**Fig. 5:**
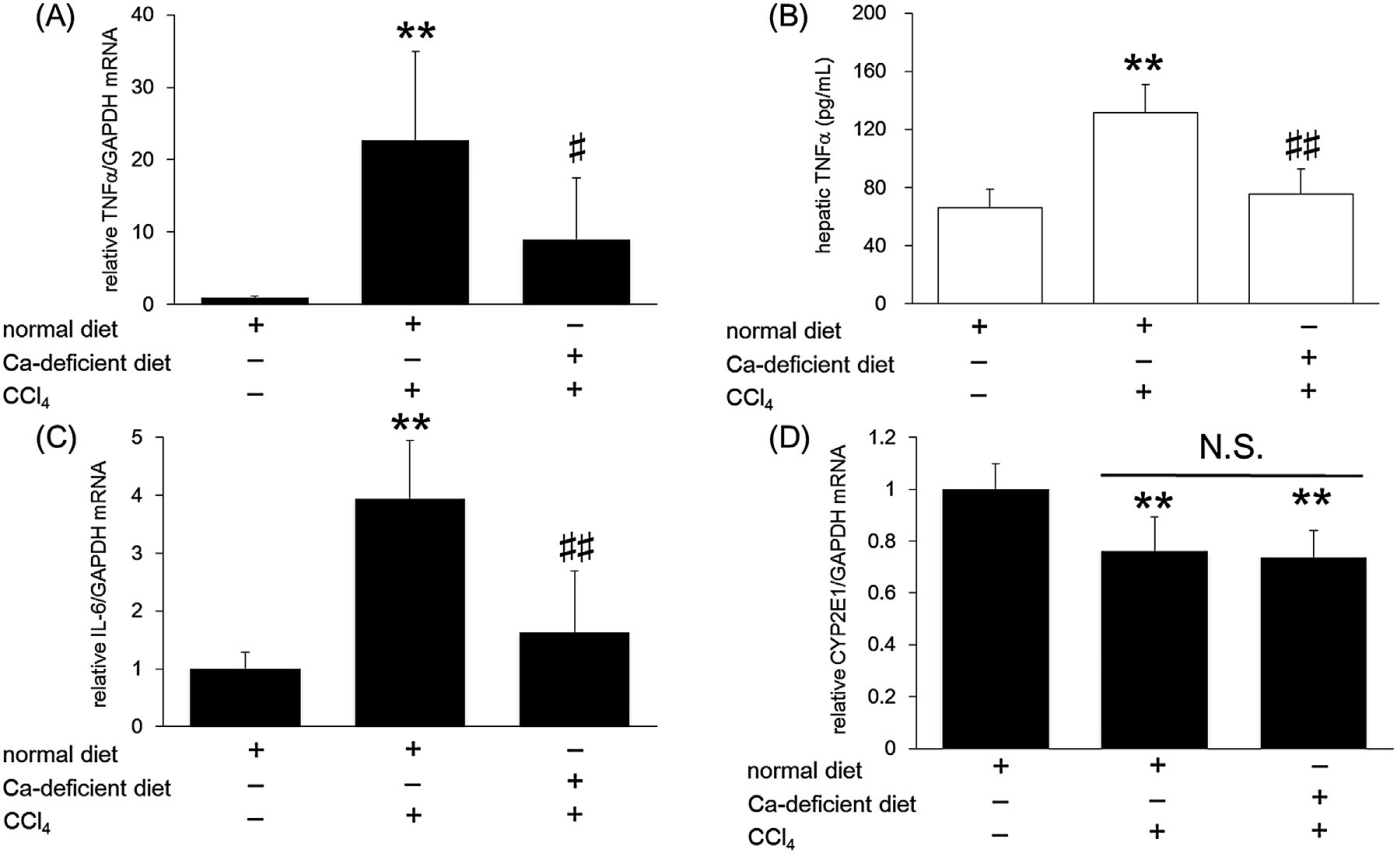
Effect of Ca-deficient diet on CCl_4_-induced liver inflammation and CYP induction. Mice were fed a normal diet or a Ca-deficient diet from 4 weeks of age for a period of 4 weeks. At 8 weeks of age, the mice were injected intraperitoneally with 2 g/kg CCl_4_. Twenty four hours after CCl_4_ injection, total RNA and total protein were isolated from the mice livers. Quantitative RT-PCR analysis and ELISA were performed. The amount of quantified target mRNA was normalized against *GAPDH mRNA*. (A), (C), and (D) indicate *TNFα*, *IL-6*, and *CYP2E1*, respectively. (B) indicates hepatic TNF-α level. Data indicate mean ± S.D. for 6–9 mice. ^**^P < 0.01 versus control, ^#^*P < 0.05* and ^##^*P < 0.01* versus normal diet + CCl_4_ group. N.S.: not significant.

It has been reported that inflammation plays an important role in CCl_4_-induced liver injury. In order to confirm involvement of Ca in this response, we determined the mRNA levels of inflammatory cytokines. As shown in Fig. 5, CCl_4_ injection increased both tumor necrosis factor-α *(TNF-α)* (A) and interleukin-6 *(IL-6)* (C) *mRNA* levels. Mice on a Ca-deficient diet showed a decrease in some, but not all, of these parameters. In addition, the protein levels of TNF-α (B) showed a similar trend. In parallel with the measurement of inflammatory cytokines, we evaluated *CYP2E1 mRNA* expression (D). CCl_4_ injection significantly decreased the levels of *CYP2E1 mRNA* in both feeding groups, but no marked differences between them were observed.

## 4 Discussion

Our current study demonstrates that a Ca-deficient diet attenuates CCl_4_-induced hepatotoxicity, but does not decrease renal toxicity. Findings from other researchers and from our previous investigation demonstrated that CCl_4_ induced severe hepatotoxicity and renal toxicity [15, 16, 17]. In the current study, hypocalcemia-induced attenuation of CCl_4_ toxicity is only observed in hepatic biochemical analysis (ALT and AST), while the levels of plasma markers of renal injury (creatinine and BUN) remained unchanged. As a Ca-deficient diet significantly decreased plasma Ca levels, this suggests that plasma Ca might preferentially effect change in the liver.

CCl_4_ is metabolized to its active form by CYPs including CYP2E1 and CYP2B family [18, 19, 20]. In fact, pretreatment with a CYP2E1 inhibitor, like an antibody or a natural product (*Antrodia camphorata*) attenuates CCl_4_-induced hepatotoxicity [21, 22, 23, 24]. In addition, Wong et al. reported that the CYP2E1 KO mouse is resistant to CCl_4_ toxicity [4]. Although CYP2E1 is also expressed in the kidney, the level of expression is much lower than that in the liver [25, 26]. We hypothesized that different CYP2E1 levels may be one cause for the relatively higher hepatic sensitivity to CCl_4_ toxicity. However, despite the markedly decreased levels of *CYP2E1* mRNA upon CCl_4_ injection, no significant difference in these levels was observed between the normal diet and the Ca-deficient diet groups. Thus, Ca-deficient diet does not seem to be involved in altering CYP induction.

CCl_4_ is widely used to investigate hepatic injury associated with oxidative stress and free radicals. The reactive oxygen species induced by CCl_4_ not only cause direct tissue damage, but also initiate inflammation through the activation of various cytokines [24, 27, 28, 29]. Oxidative stress has been postulated to be a major molecular mechanism in acute liver injury induced by CCl_4_ [2, 30, 31]. Increased TBA, a lipid peroxidative product of cell membranes, was partially attenuated by feeding the mice a Ca-deficient diet. This suggests that the protective effects of a Ca-deficient diet may be partly due to counteraction of oxidative stress in acute liver injury. In addition to oxidative stress, an inflammatory response was shown to be involved in the process of CCl_4_-induced acute chemical liver injury [28, 29, 32]. In the present study, CCl_4_-intoxicated mice on a Ca-deficient diet exhibited significant reduction in the inflammatory response compared to the mice in the normal diet group, suggesting that the beneficial effect of a Ca-deficient diet may be partly due to impairment of inflammatory response caused by CCl_4_.

The extracellular plasma Ca concentration is tightly controlled by hormones and by a complex homeostatic mechanism involving fluxes of Ca between the extracellular fluid, kidney, and bones. It has been reported that CCl_4_ disrupts hepatic Ca homeostasis [33, 34]. In our study, hepatic Ca concentration is not significantly altered by a Ca-deficient diet although there is a 28 % reduction in plasma Ca levels. There has been evidence that the upregulation of cytosolic Ca concentration is a terminal event in the progression to cell death in toxic liver injury [35]. However, the present study suggests that plasma Ca might also be a candidate trigger for mediating CCl_4_-induced toxicity. Further investigations will be needed to clarify how Ca is involved in CCl_4_-induced hepatotoxicity in other phases before the terminal phase. To investigate this, we are currently working on a vitamin D3-induced hypercalcemia model.

In conclusion, we demonstrated that a Ca-deficient diet attenuates CCl_4_-induced hepatotoxicity via suppression of lipid peroxidation and inflammatory response. To our knowledge, this is the first study to provide evidence that Ca is involved in CCl_4_-induced hepatotoxicity in a mouse model, not only in the terminal phase but also in other phases. These findings may have potential application in studies of other hepatotoxic compounds.

## Declarations

### Author contribution statement

Hiroki Yoshioka: Conceived and designed the experiments; Performed the experiments; Analyzed and interpreted the data; Contributed reagents, materials, analysis tools or data; Wrote the paper.

Tsunemasa Nonogaki: Performed the experiments; Analyzed and interpreted the data; Contributed reagents, materials, analysis tools or data.

Nobuyuki Fukuishi, Satomi Onosaka: Contributed reagents, materials, analysis tools or data.

### Funding statement

This research did not receive any specific grant from funding agencies in the public, commercial, or not-for-profit sectors.

### Competing interest statement

The authors declare no conflict of interest.

### Additional information

No additional information is available for this paper.

## Figures and Tables

**Table 1:** Oligonucleotide primer sequences and PCR conditions for real-time RT-PCR.

Gene (Accession No.)	Primer sequences		PCR Product length (bp)
Sequence (5′ to 3′)	
TNFα	Forward	GAA CTT CGG GGT GAT CGG TC	84
(NM_013693)	Reverse	GTG AGG GTC TGG GCC ATA G
IL-6	Forward	GAA ATG ATG GAT GCT ACC AAA CTG	94
(NM_031168)	Reverse	TAC TCC AGG TAG CTA TGG TAC TC
CYP2E1	Forward	CAT TCC TGT GTT CCA GGA GTA CAA G	91
(NM_021282)	Reverse	GAT ACT TAG GGA AAA CCT CCG CAC
GAPDH	Forward	TGG TGA AGG TCG GTG TGA AC	98
(NM_008084)	Reverse	GTC GTT GAT GGC AAC AAT CTC C

**Table 2:** Effect of CE-2 and Ca-deficient diet on food intake, weight gain.

	Food intake (g/day)	Weight gain (g)
Normal diet (CE-2)	6.12 ± 0.69	16.69 ± 1.39
Ca-deficient diet	5.84 ± 0.81	15.93 ± 2.01

Mice were fed a normal diet or a Ca-deficient diet from 4 weeks of age for a period of 4 weeks. Food intake was calculated as g/mouse per day. Data indicate mean ± S.D. for 8–9 mice.

**Table 3:** Effect of Ca-deficient diet on creatinine and BUN levels in CCl_4_-induced toxicity.

	Creatinine (mg/dL)	BUN (mg/dL)
Control	0.34 ± 0.05	20.03 ± 2.82
Normal diet + CCl_4_	0.72 ± 0.15**	32.69 ± 10.82**
Ca-deficient diet + CCl_4_	0.71 ± 0.14**	29.79 ± 7.59*

Mice were fed a normal diet or a Ca-deficient diet from 4 weeks of age for a period of 4 weeks. At 8 weeks of age, mice were injected intraperitoneally with 2 g/kg CCl_4_. Plasma creatinine and blood urea nitrogen (BUN) were determined 24 h after injection. Data indicate mean ± S.D. for 6–9 mice.

* *P < 0.05*.

** *P < 0.01* versus control.
